# Smoking-induced risk of future cardiovascular disease is partly mediated by cadmium in tobacco: Malmö Diet and Cancer Cohort Study

**DOI:** 10.1186/s12940-019-0495-1

**Published:** 2019-06-14

**Authors:** Huiqi Li, Björn Fagerberg, Gerd Sallsten, Yan Borné, Bo Hedblad, Gunnar Engström, Lars Barregard, Eva M. Andersson

**Affiliations:** 1 Occupational and Environmental Medicine, Sahlgrenska University Hospital and Sahlgrenska Academy, University of Gothenburg, P.O. Box 414, 405 30 Gothenburg, Sweden; 2 Department of Molecular and Clinical Medicine, Wallenberg Laboratory for Cardiovascular and Metabolic Research, University of Gothenburg and Sahlgrenska University Hospital, Gothenburg, Sweden; 3 Cardiovascular Epidemiology, Department of Clinical Sciences in Malmö, Lund University and Skåne University Hospital, Malmö, Sweden

**Keywords:** Smoking, Cadmium, Cardiovascular diseases, Mediation analysis, Prospective cohort

## Abstract

**Background:**

Smoking is a strong risk factor for cardiovascular disease (CVD) and causes exposure to cadmium, which is a pro-atherosclerotic metal. Cadmium exposure has also been shown to increase the risk of CVD, even after adjustment for smoking. Our hypothesis was that part of the risk of CVD in smokers may be mediated by cadmium exposure from tobacco smoke. We examined this hypothesis in a mediation analysis, trying to assess how much of the smoking-induced CVD risk could be explained via cadmium.

**Methods:**

We used prospective data on CVD (incidence and mortality) in a Swedish population-based cohort of 4304 middle-aged men and women (the Malmö Diet and Cancer Study). Blood cadmium was analyzed in base-line samples from 1991, and clinical events were followed up for 16–19 years based on registry data. Mediation analysis was conducted to evaluate the indirect effect (via cadmium) of smoking on CVD. Survival was analyzed by the accelerated failure time (AFT) model and the Aalen additive hazard model.

**Results:**

The mean blood cadmium level in the study population was 0.43 μg/L (median 0.24 μg/L) and increased with recent and current smoking. As expected, shorter survival time (AFT model) and higher incidence rate (Aalen model) were found in current smokers for all CVD outcomes and this effect seemed to be partly mediated by cadmium. For the sum of acute myocardial infarction, bypass grafts and percutaneous coronary intervention, and death in ischemic heart disease, about half of the increased risk of such events in current smokers was mediated via cadmium, with similar results for the AFT and Aalen models.

**Conclusions:**

Cadmium plays an important role in smoking-induced CVDs. This provides evidence for mechanisms and is of importance for both individuals and policy makers.

**Electronic supplementary material:**

The online version of this article (10.1186/s12940-019-0495-1) contains supplementary material, which is available to authorized users.

## Background

Smoking is a major risk factor of cardiovascular diseases (CVD), such as coronary heart disease and stroke [1, 2]. The underlying mechanisms have been difficult to clarify as tobacco smoke causes exposure to more than 4000 compounds, including CO, nicotine, metals, and tar containing polycyclic aromatic hydrocarbons such as benzo(a)pyrene [3, 4]. Inhaled tobacco smoke penetrates biological barriers in the form of gases, particulate matter, or soluble substances, which pass into the general circulation and reach various target tissues [5]. Cadmium is one of these important exposures and smoking is a major source of cadmium exposure [6]. Cadmium levels in blood or urine are widely used for biomonitoring [7].

Cadmium is known to be associated with kidney damage, osteoporosis, cancer, and CVD [7, 8]. The effect of cadmium on CVD has been systematically reviewed, and reports have demonstrated a 36% increase in CVD risk in the highest exposure category compared to the lowest [9, 10]. Additionally, we have shown that relatively low levels of cadmium exposure found in Sweden are also associated with incident CVD [11]. A major cause of CVD is atherosclerosis, and experimental studies as well as ultrasound studies of human atherosclerotic plaques have indicated that cadmium exposure is associated with pro-atherosclerotic effects [12–16]. A previous suggestion, that the increased risk of CVD by tobacco smoking may be partly caused by cadmium in tobacco smoke, only investigated the difference in cadmium levels between smokers and nonsmokers [17]. Using mediation analysis, we have recently shown in a cross-sectional study of a middle-aged cohort that cadmium exposure explains part of the association between smoking and the prevalence of atherosclerotic plaques in the carotid arteries [18].

In the present study we postulate that cadmium exposure is part of the causal pathway linking smoking to increased risk of CVD. Accordingly, the aim of the present study is to evaluate if, and to what degree, the smoking-associated risk of CVD is mediated by cadmium. This is done by mediation analysis [19] investigating the association between smoking and blood cadmium at baseline, and incident CVD during 16–19 years of follow-up of a Swedish cohort with cadmium levels typical of European and US populations.

## Methods

### Study cohort

The study participants have been described in detail previously [20]. Briefly, they were residents of the city of Malmö, Sweden, at baseline. Both men and women born between 1923 and 1950 were invited to participate in a health examination that took place 1991–1996. A cardiovascular substudy in subjects born 1926–1946 was performed during 1991–1994, and almost all invited subjects agreed to participate [21]. Fasting blood samples were provided by 5540 out of 6103 participants. Participants with missing data on blood cadmium and smoking status were excluded, leaving a study population of 4819 participants. A re-examination was performed during 2007–2012 (*n* = 3192) and smoking history was re-assessed as previously described [22]. The current study excluded 515 participants whose self-reported smoking status differed between baseline and re-examination, leaving 4304 participants in the study.

### Smoking status

Information of smoking status was obtained from questionnaires at baseline and at re-examination, including the year of starting to smoke, the year of smoking cessation, and the daily amount of smoking. Smoking status was categorized into four groups: never smokers, long-time former smokers (previous smokers who had stopped smoking more than 5 years prior to baseline), recent former smokers (previous smokers who had stopped smoking within 5 years prior to baseline), and current smokers. This cut-off for former smokers was based on previous reports showing that 5 years after cessation, the cardiovascular risk related to smoking is largely eliminated [23–25]. Information of pack-years (the number of packs of cigarettes smoked per day multiplied by the number of years the person has smoked) was available for current smokers, never smokers (0 pack-years) and part of the former smokers.

### Data and sample collection

Baseline status of cardiovascular risk factors was obtained through questionnaire, including information regarding smoking, alcohol consumption, socioeconomic status, education, physical activity, medical history and medication. Height, weight, and waist circumference were recorded, and body mass index was calculated. Blood pressure was measured after participants rested in the supine position for 10 min. Overnight fasting blood samples were obtained by standard procedures at Malmö University Hospital. High-density lipoprotein cholesterol, low-density lipoprotein cholesterol, hemoglobin A1c, C-reactive protein, and whole blood glucose were analyzed as previously reported [16].

Erythrocytes were collected by centrifuging whole blood in heparin tubes, and then kept at -80 °C in cryotubes (Nunc®; Sigma-Aldrich, Darmstadt, Germany) until analysis. Cadmium and lead in erythrocytes were analyzed by inductively coupled plasma mass spectrometry, as previously described [16, 26]. Blood cadmium was then estimated by multiplying the cadmium concentration by hematocrit. The limit of detection was 0.02 μg/L for cadmium and 0.16 μg/L for lead. Based on 50 duplicate samples, the coefficient of variation for blood cadmium was calculated to be 9.6%, and the reliability ratio was calculated to be 0.994.

### Cardiovascular outcomes

All participants were followed until 31 December 2010, except for 30 subjects who emigrated during the study period. The incidences of cardiovascular events were obtained from the Swedish National Hospital Discharge Register and Cause of Death Register, the Malmö Stroke Register, and the Swedish Coronary Angiography and Angioplasty Registry. Cardiovascular outcomes were defined based on the *International Classification of Diseases,* 9th or 10th Revision (ICD-9 or ICD-10), and the end points were as follows (ICD codes):

- Acute coronary event, defined as acute myocardial infarction (ICD-9: 410; ICD-10: I21) or death due to ischemic heart disease (ICD-9: 412 or 414; ICD-10: I22, I23, or I25)
- Major adverse coronary event (MACE), including acute coronary event, coronary artery bypass graft (CABG), and percutaneous coronary intervention (PCI). The last two, CABG and PCI, were identified from national registries
- Stroke (ICD-9: 430, 431, 434, 436; ICD-10: I60, I61, I63– I64)
- Cardiovascular mortality (ICD-9: 390–459; ICD-10: I00– I99)
- All-cause mortality.

The first three outcomes above included both fatal and non-fatal cases. For each specific outcome, only the first event was considered; for example, a participant who had his or her first stroke event before baseline would be excluded from the analysis of stroke. A participant could contribute to multiple outcomes, for example first suffering a stroke and later having a cardiovascular death.

### Statistical analysis

The survival times until the specific cardiovascular event or censoring (due to the end of follow-up, or to migration or death) were used as outcomes, smoking status as exposure, and blood cadmium as mediator in this study. The exposure to smoking was defined as a categorical variable with four groups, and the “Never smoker” group was considered as the reference group throughout the analyses. The adjustments included were selected a priori based on the scientific literature on potential confounders: age, gender, waist circumference, education (low/high), physical activity (low/high), blood lead, ratio of total cholesterol/high density lipoprotein, hemoglobin A1c, and C-reactive protein.

Previous papers [27–29] have discussed the statistical method for mediation analysis in the context of survival analysis and suggested that the Cox proportional hazard model is not suitable for mediation analysis, given that it is not mathematically consistent when the mediator is included in, vs. excluded from, the model. Several alternative methods have been suggested for mediation analysis in survival data. One suggestion is to use the accelerated failure time (AFT) model, where results are interpreted as the fold change in survival time [30]; the other is to use the Aalen additive hazard model, where the results are interpreted as the number of additional events per person-time [27]. In this study, the analyses were performed using both the AFT model (assuming a Weibull distribution) and the Aalen additive hazard model (regression coefficients are assumed constant over time) with age on the time axis. The potential interaction between exposure and mediator was also tested in the models. A linear model was used to estimate the association between the exposure and the mediator.

The mediator, blood cadmium, was used in its original form in the mediation analysis based on biological plausibility. We also performed a sensitivity analysis where blood cadmium was log-transformed.

We also performed the analysis with pack-years as exposure instead of smoking categories.

A sensitivity analysis was performed by including participants with inconsistent smoking status (*n* = 515) in order to examine if potential misclassification of smoking status would change our results.

In addition, the impact of the measurement error in blood cadmium was examined. We used the results of the direct and indirect effects for MACE in the current smoker group as an example, and calculated how biased the results would be, given our reliability ratio [31].

A sensitivity analysis for unmeasured mediator-outcome confounding was performed. We used the results of the direct and indirect effects for MACE in the current smoker group as an example, and calculated how biased the results would be, given certain effects of the unmeasured confounder on the mediator and the outcome [32]. Briefly, the sensitivity analysis was made for the situation where the unmeasured confounder increases cadmium (α_3_ > 0) and shortens survival time (exp(β_5_) < 1, AFT model) or increases the hazard (β_5_ > 0, Aalen model), based on the notation (β_0_ + β_1_*Exposure + β_2_*Mediator + β_3_*(Exposure*Mediator) + β_4_*Covariates + β_5_*Unmeasured confounder) for the outcome regression model, and (α_0_+ α_1_*Exposure + α_2_*Covariates + α_3_*Unmeasured confounder) with residual variance σ^2^_M_ for the linear mediator regression model. The parameters used in this analysis (values for α_1_ and σ^2^_M_) were derived from the models.

Statistical analyses were conducted in SAS 9.4 (SAS Institute, Cary, NC, USA) and R 3.4.0 (R core team) with packages of OIsurv (David M Diaz), timereg (Thomas H. Scheike), and mvtnorm (Alan Genz). Confidence intervals for the direct effects were estimated based on standard errors from the AFT and Aalen models, and confidence intervals for the indirect and total effects were estimated using simulation with 100,000 repeats, as described [27, 30]. Proportion mediated was calculated as the ratio between the indirect effect and the total effect in the recent former smoker group and the current smoker group.

## Results

Number of participants, number of events, person-years, and event rates in each smoking group are listed in Table 1. The general characteristics at baseline are presented in Table 2. The mean blood cadmium level in the study population was 0.43 μg/L (median 0.24 μg/L). Blood cadmium increased with recent and current smoking (Jonckheere’s trend test *P* < 0.001). The age distribution among the four groups was almost identical, with an average of 58 years. The highest percentage of women (70%) was found in the never-smoking group, while the male to female ratio was similar in the other three groups. C-reactive protein increased with recent and current smoking. The mean blood lead level in the study participants was 28.6 μg/L (median 25.4 μg/L). It increased with recent and current smoking, but much less than blood cadmium did.

**Table 1:** Number of subjects, number of events, person-years, and events/person-year rate in each smoking group

Outcome	Smoking status	n	Events	Person-years	Events/thousand person-years
Acute coronary event	Never smoker	1884	119	32,001	3.7
	Stopped > 5 years	1225	101	20,309	5.0
	Stopped <= 5 years	278	26	4424	5.9
	Current smoker	840	101	12,598	8.0
Major adverse cardiac event	Never smoker	1879	145	31,763	4.6
	Stopped > 5 years	1218	132	19,925	6.6
	Stopped <= 5 years	275	36	4282	8.4
	Current smoker	837	120	12,423	9.7
Stroke	Never smoker	1887	128	32,087	4.0
	Stopped > 5 years	1240	75	20,537	3.7
	Stopped <= 5 years	289	23	4568	5.0
	Current smoker	843	79	12,672	6.2
Cardiovascular mortality	Never smoker	1895	79	32,818	2.4
	Stopped > 5 years	1253	71	21,170	3.4
	Stopped <= 5 years	293	19	4789	4.0
	Current smoker	853	84	13,137	6.4
All-cause mortality	Never smoker	1895	265	32,818	8.1
	Stopped > 5 years	1253	230	21,170	10.9
	Stopped <= 5 years	293	67	4789	14.0
	Current smoker	853	304	13,137	23.1

**Table 2:** Baseline characteristics and potential cardiovascular risk factors of the study participants (n = 4304)

Characteristics and risk factors	Total	Never smokers	Long time former smokers	Recent former smokers	Current smokers
Blood cadmium (μg/L) [Median (p5 - p95)^1^]	0.24 (0.10–1.52)	0.20 (0.089–0.46)	0.22 (0.10–0.50)	0.36 (0.15–1.11)	1.00 (0.22–2.46)
Age (year) [Median (p5 - p95)^1^]	58 (48–66)	58 (48–66)	58 (48–66)	57 (48–66)	57 (48–66)
Women [n (%)]	2540 (59%)	1329 (70%)	586 (47%)	152 (52%)	473 (56%)
Waist (cm) [Median (p5 - p95)^1^]	84 (65–106)	82 (65–103)	87 (66–110)	87 (66–110)	83 (64–105)
Low education [n (%)]	1990 (47%)	853 (45%)	545 (44%)	151 (52%)	441 (52%)
Low physical activity [n (%)]	984 (24%)	427 (23%)	246 (20%)	72 (25%)	241 (29%)
High alcohol intake [n (%)]	154 (3.6%)	35 (1.9%)	55 (4.4%)	11 (3.8%)	53 (6.3%)
Total cholesterol / HDL [Median (p5 - p95)^1^]	4.5 (2.8–7.6)	4.4 (2.8–7.3)	4.5 (2.8–7.3)	4.7 (2.8–8.2)	4.8 (2.8–8.0)
HbA1c (mmol/mol) [Median (p5 - p95)^1^]	4.8 (4.1–5.8)	4.7 (4.1–5.6)	4.7 (4.1–5.8)	4.9 (4.1–5.8)	5.0 (4.3–6.0)
CRP (mg/L) [Median (p5 - p95)^1^]	1.4 (0.3–8.3)	1.2 (0.3–6.8)	1.3 (0.3–8.2)	1.6 (0.4–8.9)	1.8 (0.3–11.5)
Blood lead (μg/L) [Median (p5 - p95)^1^]	25.4 (12.7–54.4)	23.1 (12.1–48.9)	26.3 (12.4–51.8)	27.0 (13.1–51.9)	29.4 (14.5–69.4)

1) p5 – p95: 5th percentile to 95th percentile

Results of mediation analysis based on the accelerated failure time model are presented in Table 3. Current smokers had shorter survival time compared to never smokers for all outcomes in this study (total effect, Table 3). In recent former smokers, the effect estimates also suggested a shorter survival time than in never smokers with varied *p* values. Indirect effects with confidence intervals excluding null (1.00 as null) were found in current smokers regarding MACE and all-cause mortality. For almost all outcomes except MACE in current smokers, the direct effects were stronger than the indirect effects. The proportion mediated among current smokers varied from 8% (cardiovascular mortality) to 48% (MACE), with an average of 22%. For recent former smokers, the mean proportion mediated was 11%. These results suggest that cadmium exposure is an important mediator of cardiovascular risk among current smokers, contributing approximately one-fifth of the total risk.

**Table 3:** Mediation analysis with the aft model, data from 4304 subjects

Aimed event	Smoking status	Direct effect^1^		Indirect effect^1^		Total effect^1^		PM^2^
		estimate	95% CI^3^	estimate	95% CI^3^	estimate	95% CI^3^	
Acute coronary event	Never smoker							
	Long time former smoker	1.00	0.85, 1.19	1.00	0.99, 1.00	1.00	0.85, 1.19	
	Recent former smoker	0.83	0.64, 1.09	0.99	0.96, 1.02	0.83	0.63, 1.08	5%
	Current smoker	0.75^a^	0.60, 0.94	0.96	0.84, 1.09	0.72^a^	0.60, 0.86	12%
Major adverse coronary event	Never smoker							
	Long time former smoker	0.93	0.79, 1.09	1.00	0.99, 1.00	0.92	0.79, 1.08	
	Recent former smoker	0.85	0.66, 1.10	0.96^a^	0.93, 0.99	0.82	0.63, 1.06	21%
	Current smoker	0.86	0.70, 1.07	0.85^a^	0.75, 0.96	0.73^a^	0.62, 0.87	48%
Stroke	Never smoker							
	Long time former smoker	1.17	0.95, 1.43	1.00	0.99, 1.00	1.16	0.95, 1.43	
	Recent former smoker	0.89	0.65, 1.21	0.98	0.94, 1.02	0.87	0.64, 1.18	15%
	Current smoker	0.81	0.63, 1.06	0.93	0.79, 1.08	0.75^a^	0.61, 0.92	25%
Cardiovascular mortality	Never smoker							
	Long time former smoker	0.98	0.94, 1.02	1.00	1.00, 1.00	0.98	0.94, 1.02	
	Recent former smoker	0.97	0.9, 1.05	1.00	0.99, 1.00	0.97	0.90, 1.04	0%
	Current smoker	0.90^a^	0.85, 0.95	0.99	0.95, 1.02	0.89^a^	0.85, 0.93	8%
All-cause mortality	Never smoker							
	Long time former smoker	0.98	0.95, 1.01	1.00	1.00, 1.00	0.98	0.95, 1.00	
	Recent former smoker	0.95	0.91, 1.00	0.99	0.99, 1.00	0.95^a^	0.91, 0.99	20%
	Current smoker	0.88^a^	0.85, 0.91	0.97^a^	0.96, 0.99	0.86^a^	0.84, 0.88	20%

^1^The effects are presented as fold change of survival time; ^2^PM: proportion mediated (presented for recent former smokers and current smokers); ^3^95% CI: 95% confidence interval; 4) ^a^denotes *P* < 0.05

Results of mediation analysis based on the Aalen additive hazard model are presented in Table 4. Incidence rates in current smokers were higher than in never smokers for all outcomes (total effect, Table 4). Recent former smokers had higher incidence rates compared to never smokers regarding MACE and all-cause mortality. The confidence intervals of the indirect effects all covered null (0 as null), however, the indirect effect of MACE for current smokers was borderline significant (but significant in the AFT model). The proportion mediated among current smokers varied from 3% (cardiovascular mortality) to 58% (MACE), with an average of 20%. For recent former smokers, the mean proportion mediated was 8%. As expected, the Aalen additive hazard model provided similar results to the AFT model, suggesting that current smoking is an important risk factor for various cardiovascular outcomes, and cadmium is an important pathway for such effects.

**Table 4:** Mediation analysis with the Aalen additive hazard model, data from 4304 subjects

Aimed event	Smoking status	Direct effect^1^		Indirect effect^1^		Total effect^1^		PM^2^
		estimate	95% CI^3^	estimate	95% CI^3^	estimate	95% CI^3^	
Acute coronary event	Never smoker							
	Long time former smoker	0.25	−0.73, 1.22	0.0044	− 0.030, 0.044	0.25	− 0.72, 1.23	
	Recent former smoker	1.80	−0.13, 3.73	0.044	−0.24, 0.33	1.84	−0.088, 3.77	2%
	Current smoker	2.34^a^	0.51, 4.17	0.18	−0.95, 1.31	2.52^a^	1.13, 3.90	7%
Major adverse coronary event	Never smoker							
	Long time former smoker	0.79	−0.33, 1.92	0.036	−0.013, 0.11	0.83	−0.29, 1.96	
	Recent former smoker	1.96	−0.23, 4.15	0.37	−0.015, 0.78	2.33^a^	0.16, 4.51	16%
	Current smoker	1.10	−0.94, 3.14	1.50	−0.030, 3.05	2.60^a^	1.07, 4.13	58%
Stroke	Never smoker							
	Long time former smoker	−0.56	−1.45, 0.32	0.0051	−0.027, 0.043	−0.55	−1.44, 0.33	
	Recent former smoker	0.82	−0.89, 2.52	0.046	−0.20, 0.30	0.86	−0.85, 2.59	5%
	Current smoker	1.10	−0.61, 2.81	0.18	−0.79, 1.16	1.28^a^	0.028, 2.53	14%
Cardiovascular mortality	Never smoker							
	Long time former smoker	0.49	−0.29, 1.26	0.0019	−0.031, 0.037	0.49	−0.29, 1.26	
	Recent former smoker	0.81	−0.62, 2.24	0.017	−0.22, 0.26	0.82	−0.57, 2.22	2%
	Current smoker	2.57^a^	0.97, 4.17	0.065	−0.89, 1.01	2.63^a^	1.40, 3.88	3%
All-cause mortality	Never smoker							
	Long time former smoker	1.29	−0.12, 2.70	0.059	−0.012, 0.17	1.35	−0.055, 2.76	
	Recent former smoker	2.95^a^	0.28, 5.62	0.52	−0.010, 1.09	3.48^a^	0.86, 6.11	15%
	Current smoker	8.26^a^	5.24, 11.3	2.08	−0.062, 4.21	10.3^a^	8.05, 12.6	20%

^1^ The effects are presented as number of additional cases per 1000 person-years; ^2^PM: proportion mediated (presented for recent former smokers and current smokers); ^3^ 95% CI: 95% confidence interval; 4) ^a^denotes *P* < 0.05

The results from the analysis with log-transformed blood cadmium are shown in Additional file 1: Table S1a and b. In general, the analyses with log-transformed blood cadmium showed smaller direct and larger indirect effects, in comparison to analyses based on non-transformed blood cadmium values.

The analyses by using pack-years of smoking as exposure included 3523 individuals with complete data. The results in Additional file 1: Table S2a and b showed stronger indirect effects, with none of the confidence intervals covering the null value (null value is 1 for the AFT model and 0 for the Aalen model).

The results of the analysis including participants with inconsistent smoking status were highly consistent with the results from the main study population (Additional file 1: Table S3a and b).

The sensitivity analysis for measurement error in blood cadmium showed that, the bias introduced by measurement error of blood cadmium was negligible based on the high reliability ratio. For example, the estimates for the direct and indirect effect for MACE in the current smoker group would change by less than 1% (data not shown).

The sensitivity analysis for unmeasured confounder indicated that an unmeasured confounder needs to have rather strong effects on both blood cadmium and CVD risk in order to induce a large bias on the direct and indirect effects (Additional file 1: Table S4). For example, if being exposed to an unmeasured confounder could lead to an increase of blood cadmium by 0.3 μg/L, and could lead (directly) to 92% shorter survival time, this unmeasured confounder could nullify the indirect effect found by the AFT model regarding MACE in this study. Such a hypothetical effect of the unmeasured confounder on cadmium would be very strong. Also, the effect of the unmeasured confounder on CVD risk would need to constitute about half of the direct effect of smoking. To nullify the indirect effect found by the Aalen model, it would take an unmeasured confounder which would lead to an increase of blood cadmium by 0.4 μg/L and cause (directly) 0.5 extra incidence of MACE per 1000 person-year (about half of the direct effect of smoking).

## Discussion

This study used mediation analysis to analyze the possible pathway linking smoking to CVD and mortality. The results consistently indicated that part of the effect of smoking is mediated through cadmium. This indirect effect of cadmium contributed about one-fifth of the smoking-induced risk of CVD.

This mediation study is based on the pre-defined smoking-cadmium-CVD pathway. The results are meaningful only when the pathway is valid. One concern is that cadmium might only be a proxy for other toxins in tobacco. However, available data do not support such a concept. In a meta-analysis [9], a subgroup analysis of never smokers in nine published studies which included CVD, coronary heart disease, heart failure, stroke and peripheral artery disease, there was a pooled relative event risk of 1.27 (95% CI: 0.97, 1.67) per change in cadmium levels. In the Strong Heart Study there was a relative CVD risk of 1.26 (1.00, 1.58) in 80th vs 20th percentiles of urinary cadmium in never smokers [33]. In this cohort, the hazard ratio of MACE was 2.2 (1.0, 4.6) in never smokers in 4th vs 1st quartiles of blood cadmium [11]. Therefore, the pathway of smoking-cadmium-CVD risk should be valid and it is unlikely that cadmium is merely a proxy for other compounds in tobacco smoke.

The results of the mediation analysis based on the AFT model should be interpreted as the fold change of survival time compared to the never smoker group. For example, current smokers on average had 27% shorter survival time until their first MACE compared to never smokers, and 15% could be attributed to cadmium from smoking (Table 3). The results with the Aalen additive hazard model can be interpreted as the additional event rate in smokers compared to never smokers. Current smokers would have 2.60 extra MACE per 1000 person-year compared to never smokers, and 1.50 of these would be due to cadmium from smoking (Table 4). Not many of the indirect effects are statistically significant (with their 95% confidence intervals covering null), and this is probably due to limited statistical power. This issue could be better addressed by longer follow-up time, and/or larger study population size. Nevertheless, the results by the two models demonstrated from different angles that cadmium in smoking tobacco is a very likely mechanism contributing to smoking-induced CVD.

The results of this study provide quantitative assessment for effects of certain interventions. However, the potential intervention depends on which part of the effect is considered. 1) When discussing the total effect, the intervention would be “never smoke”. Such an intervention is important on a population level. 2) The direct effect obtained (from the AFT and the Aalen models) is the natural direct effect. In this study it would mean that if the smokers had the same level of blood cadmium as never smokers (they had cadmium from other sources), the direct effect estimates what CVD risk they would face compared to never smokers. As the results indicated, smokers would still have an increased CVD risk, even if the tobacco had been free of cadmium. The remaining CVD risk by smoking is probably caused by other toxicants in smoking. It should be noted that cadmium from other sources are not relevant here because it was not assumed that the smokers had zero blood cadmium, instead we assumed they would have the same level as never smokers. 3) When focusing on the indirect effect, where the effect of the smoking-cadmium-CVD risk pathway was estimated, the comparison would be between the smokers in the real world where smoking tobacco contains cadmium and smokers if they had smoked cadmium-free tobacco (therefore the level of blood cadmium was counterfactually set to the level which it naturally would be if they did not smoke). The intervention could be defined as “to ban cadmium in tobacco” (or to decrease cadmium pollution, which is causing cadmium in tobacco). As our results showed, such an intervention was estimated to reduce the CVD risk.

Since not all the participants provided information at the re-examination, some participants may have been misclassified regarding smoking group. To investigate if such potential misclassification could influence the results, we performed a sensitivity analysis where the 515 participants with inconsistent self-reported smoking status were also included in the models. The results were similar to the ones obtained for the restricted study population. Therefore, such potential misclassification should not have affected our results.

The non-confounding assumption in mediation analysis should control for: 1) exposure-outcome confounding, 2) mediator-outcome confounding, 3) exposure-mediator confounding, and one must further assume that, 4) none of the mediator-outcome confounders are affected by the exposure [34]. The co-exposures from smoking would be potential mediator-outcome confounders if they had common sources with cadmium other than smoking. An example would be simultaneous exposure to cadmium and lead in food. It was noted that in the never smoker group blood cadmium and blood lead were associated. Therefore blood lead was included in the adjustment. Unfortunately the information on other co-exposures were not available. Therefore, we performed a sensitivity analysis for unmeasured mediator-outcome confounders, and it indicated that an unmeasured confounder needs to be very influential on both cadmium and CVD risk to induce a substantial bias. Based on the knowledge of sources of cadmium exposure and of risk factors for CVD, we consider such strong unmeasured confounders not to be realistic.

Blood cadmium was non-transformed in the main analyses in this study. Log transformation was not considered as the best choice because it is not plausible to assume that a fold change of blood cadmium at very low cadmium levels (e.g., from 0.01 to 0.02 μg/L) would increase CVD risk to the same extent as a similar fold change at normal or high blood cadmium levels (e.g., from 0.4 to 0.8 μg/L). A linear association is biologically more plausible. However, we did perform a sensitivity analysis where blood cadmium was log-transformed. The results tended to increase the indirect effect of cadmium, which therefore strengthened our conclusion that part of the effect of smoking on CVD risk is mediated via cadmium.

Regarding external validity, the blood cadmium levels found in this study (mean 0.43 μg/L) are typical for middle-aged European and US populations [35, 36].

To our knowledge, this is the first study using mediation analysis to investigate the mechanism of smoking-induced cardiovascular risk linked to cadmium in a prospective cohort. As previously discussed, mediation analysis is a powerful tool to disentangle direct and indirect effects, which can provide insights on mechanisms [19]. The present results support the hypothesis that the cadmium content in tobacco smoke can cause CVD, raising questions about pathophysiology. In a population perspective, CVDs are known to be caused by atherosclerosis which leads to the formation of plaques in arterial vessel walls. The available body of data indicates pro-atherosclerotic effects of cadmium exposure as shown in experimental and human studies [12–16]. In addition, we have recently shown that the smoking-associated occurrence of atherosclerotic plaques in carotid arteries is partly mediated by cadmium exposure [18]. However, asymptomatic atherosclerotic plaques are very common in older people. In principle, coronary events and stroke occur only if these plaques become vulnerable, rupture, and cause thrombosis [37], and indeed, there are indications that cadmium increases plaque vulnerability. In symptomatic carotid plaques, compared to blood, there is a 50-fold increase in cadmium levels, and the cadmium concentration is the highest in the part of the plaque that usually ruptures [38]. In such plaques, there is a positive association between blood cadmium concentration and the degree of inflammation in those sections of the plaque with the most frequent ruptures [39]. A possible mechanism behind the role of cadmium in CVD is that cadmium depletes glutathione and protein-bound sulfhydryl groups, leading to increased generation of reactive oxygen species, which could in turn result in various adverse effects including lipid peroxidation, DNA damage, membrane damage, inflammation, etc., and thereby maintain a vicious circle eventually leading to plaque formation and clinical events [40]. Taken together, this data provides a plausible mechanistic explanation for the finding of an indirect cadmium-related effect on smoking-associated risk of CVD.

There are several limitations to this study. Firstly, smoking status was self-reported, which can lead to misclassification due to memory error. Detailed information on smoking burden such as pack years was not available for all participants. Secondly, exposure through passive smoking was not included in the model, which may have influenced the results to some extent [41]. Thirdly, urinary cadmium concentrations were not available as a long term measure of cadmium exposure. However, in a study based upon NHANES data it was found that blood cadmium concentrations showed highly significant correlations with smoking duration, smoking dose and pack-years in former smokers with no dramatic differences compared to similar analysis with urine cadmium levels [42]. Hence, we believe that blood cadmium has been a sufficient measure of smoking exposure. Fourthly, for some of the CVD outcomes (e.g., cardiovascular mortality), there were few cases in the study, which led to limited statistical power to detect a true effect.

## Conclusions

In summary, we have found that cadmium exposure seems to be an important pathway for smoking-induced CVD. This opens up new insights into disease processes and could be important both for individuals at risk and policy makers. The results also clearly demonstrate that smoking should be avoided and that cadmium exposure in the population should be minimized.

## Additional file

Additional file 1: **Table S1a.** Mediation analysis with AFT model, with log-transformed blood cadmium, data from *n* = 4304 subjects. The effects are presented as fold change of survival time. **Table S1b.** Mediation analysis with Aalen model, with log-transformed blood cadmium, data from n = 4304 subjects. The effects are presented as number of additional cases per 1000 person-years. **Table S2a.** Mediation analysis with AFT model, with pack-years as exposure, data from *n* = 3523 subjects. The effects are presented as fold change of survival time per 1 pack-year. **Table S2b.** Mediation analysis with Aalen model, with pack-years as exposure, data from n = 3523 subjects. The effects are presented as number of additional cases per 1000 person-years per 1 pack-year. **Table S3a.** Mediation analysis with AFT model including 515 participants with inconsistent reported smoking status, data from *n* = 4819 subjects. The effects are presented as fold change of survival time. **Table S3b.** Mediation analysis with Aalen model including 515 participants with inconsistent reported smoking status, data from n = 4819 subjects. The effects are presented as number of additional cases per 1000 person-years. **Table S4.** Sensitivity analysis for unmeasured mediator-outcome confounder. The results of direct effects and indirect effects for major adverse cardiac event in current smoker group were taken as an example here. The analysis is made for the situation where the unmeasured confounder increases cadmium (α3 > 0) and shortens survival time (exp(β5) < 1, AFT) or increases the hazard (β5 > 0, Aalen). Corrected estimates of direct and indirect effects are listed below, given certain values of the effect of the unmeasured confounder on cadmium (α3), the effect of the confounder on survival (exp(β5) for AFT model and β5 for Aalen model). (DOCX 57 kb)

## Data Availability

The data that support the findings of this study are available from Gothenburg University, but restrictions apply to the availability of these data, which were used under license for the current study, and so are not publicly available. Data are however available from the authors upon reasonable request and with permission of Gothenburg University.

## Abbreviations

AFT: Accelerated failure time
CVD: Cardiovascular disease
ICD: International Classification of Diseases
MACE: Major adverse coronary event
