# Supplementary material for: Smoking-induced risk of future cardiovascular disease is partly mediated by cadmium in tobacco: Malmö Diet and Cancer Cohort Study
Source: Environ Health. 2019 Jun 14;18:56. doi: 10.1186/s12940-019-0495-1 (PMC6570857; doi:10.1186/s12940-019-0495-1)
Supplement: Supplementary file 1 — Table S1a. Mediation analysis with AFT model, with log-transformed blood cadmium, data from n = 4304 subjects. The effects are presented as fold change of survival time. Table S1b. Mediation analysis with Aalen model, with log-transformed blood cadmium, data from n = 4304 subjects. The effects are presented as number of additional cases per 1000 person-years. Table S2a. Mediation analysis with AFT model, with pack-years as exposure, data from n = 3523 subjects. The effects are presented as fold change of survival time per 1 pack-year. Table S2b. Mediation analysis with Aalen model, with pack-years as exposure, data from n = 3523 subjects. The effects are presented as number of additional cases per 1000 person-years per 1 pack-year. Table S3a. Mediation analysis with AFT model including 515 participants with inconsistent reported smoking status, data from n = 4819 subjects. The effects are presented as fold change of survival time. Table S3b. Mediation analysis with Aalen model including 515 participants with inconsistent reported smoking status, data from n = 4819 subjects. The effects are presented as number of additional cases per 1000 person-years. Table S4. Sensitivity analysis for unmeasured mediator-outcome confounder. The results of direct effects and indirect effects for major adverse cardiac event in current smoker group were taken as an example here. The analysis is made for the situation where the unmeasured confounder increases cadmium (α3 > 0) and shortens survival time (exp(β5) < 1, AFT) or increases the hazard (β5 > 0, Aalen). Corrected estimates of direct and indirect effects are listed below, given certain values of the effect of the unmeasured confounder on cadmium (α3), the effect of the confounder on survival (exp(β5) for AFT model and β5 for Aalen model). (DOCX 57 kb) [file 12940_2019_495_MOESM1_ESM.docx]

Additional file 1: Table S1a. Mediation analysis with AFT model, with log-transformed blood cadmium, data from n=4304 subjects. The effects are presented as fold change of survival time.

| Aimed event | Smoking status | Direct effect | | Indirect effect | | Total effect | |
| --- | --- | --- | --- | --- | --- | --- | --- |
|  |  | estimate | 95% CI | estimate | 95% CI | estimate | 95% CI |
| Acute coronary event | Never smoker | --- | --- | --- | --- | --- | --- |
|  | Long time former smoker | 1.03 | 0.87, 1.22 | 0.98 | 0.97, 1.00 | 1.02 | 0.86, 1.20 |
|  | Recent former smoker | 0.92 | 0.70, 1.22 | 0.93 | 0.86, 1.01 | 0.86 | 0.67, 1.11 |
|  | Current smoker | 0.84 | 0.66, 1.08 | 0.85 | 0.72, 1.02 | 0.72a | 0.60, 0.86 |
| Major adverse coronary event | Never smoker | --- | --- | --- | --- | --- | --- |
|  | Long time former smoker | 0.97 | 0.83, 1.14 | 0.97a | 0.95, 0.99 | 0.95 | 0.81, 1.11 |
|  | Recent former smoker | 0.94 | 0.73, 1.22 | 0.89a | 0.82, 0.95 | 0.84 | 0.66, 1.06 |
|  | Current smoker | 0.95 | 0.75, 1.20 | 0.76a | 0.64, 0.90 | 0.72a | 0.61, 0.85 |
| Stroke | Never smoker | --- | --- | --- | --- | --- | --- |
|  | Long time former smoker | 1.15 | 0.94, 1.41 | 0.98 | 0.96, 1.00 | 1.13 | 0.93, 1.38 |
|  | Recent former smoker | 0.93 | 0.68, 1.28 | 0.93 | 0.84, 1.02 | 0.87 | 0.65, 1.17 |
|  | Current smoker | 0.88 | 0.66, 1.18 | 0.85 | 0.69, 1.04 | 0.75a | 0.61, 0.91 |
| Cardiovascular mortality | Never smoker | --- | --- | --- | --- | --- | --- |
|  | Long time former smoker | 0.95 | 0.81, 1.10 | 0.99 | 0.97, 1.00 | 0.93 | 0.80, 1.09 |
|  | Recent former smoker | 0.92 | 0.72, 1.17 | 0.95 | 0.88, 1.01 | 0.87 | 0.69, 1.09 |
|  | Current smoker | 0.77a | 0.62, 0.95 | 0.88 | 0.75, 1.03 | 0.67a | 0.58, 0.79 |
| All-cause mortality | Never smoker | --- | --- | --- | --- | --- | --- |
|  | Long time former smoker | 0.91 | 0.83, 1.01 | 0.99a | 0.97, 1.00 | 0.90a | 0.82, 0.99 |
|  | Recent former smoker | 0.83a | 0.71, 0.97 | 0.94a | 0.90, 0.98 | 0.78a | 0.68, 0.91 |
|  | Current smoker | 0.67a | 0.59, 0.77 | 0.87a | 0.79, 0.96 | 0.58a | 0.53, 0.64 |

1. 95% CI: 95% confidence interval; 2) a denotes *P* <0.05.

Table S1b. Mediation analysis with Aalen model, with log-transformed blood cadmium, data from n=4304 subjects. The effects are presented as number of additional cases per 1000 person-years.

| Aimed event | Smoking status | Direct effect | | Indirect effect | | Total effect | |
| --- | --- | --- | --- | --- | --- | --- | --- |
|  |  | estimate | 95% CI | estimate | 95% CI | estimate | 95% CI |
| Acute coronary event | Never smoker | --- | --- | --- | --- | --- | --- |
|  | Long time former smoker | 0.11 | -0.81, 1.03 | 0.079 | -0.038, 0.20 | 0.19 | -0.73, 1.11 |
|  | Recent former smoker | 1.22 | -0.60, 3.04 | 0.34 | -0.17, 0.86 | 1.57 | -0.25, 3.39 |
|  | Current smoker | 1.41 | -0.31, 3.13 | 0.78 | -0.37, 1.93 | 2.19a | 0.92, 3.46 |
| Major adverse coronary event | Never smoker | --- | --- | --- | --- | --- | --- |
|  | Long time former smoker | 0.51 | -0.55, 1.57 | 0.18a | 0.044, 0.33 | 0.69 | -0.37, 1.75 |
|  | Recent former smoker | 1.48 | -0.64, 3.60 | 0.78a | 0.20, 1.38 | 2.27a | 0.16, 4.38 |
|  | Current smoker | 0.65 | -1.25, 2.56 | 1.79a | 0.46, 3.13 | 2.44a | 1.02, 3.85 |
| Stroke | Never smoker | --- | --- | --- | --- | --- | --- |
|  | Long time former smoker | -0.45 | -1.32, 0.42 | 0.071 | -0.042, 0.19 | -0.38 | -1.24, 0.48 |
|  | Recent former smoker | 0.68 | -1.01, 2.37 | 0.30 | -0.18, 0.80 | 0.99 | -0.70, 2.67 |
|  | Current smoker | 0.79 | -0.86, 2.43 | 0.68 | -0.41, 1.77 | 1.46a | 0.28, 2.66 |
| Cardiovascular mortality | Never smoker | --- | --- | --- | --- | --- | --- |
|  | Long time former smoker | 0.44 | -0.29, 1.16 | 0.047 | -0.056, 0.16 | 0.48 | -0.25, 1.20 |
|  | Recent former smoker | 0.83 | -0.63, 2.28 | 0.2 | -0.23, 0.63 | 1.02 | -0.36, 2.41 |
|  | Current smoker | 1.89a | 0.35, 3.43 | 0.45 | -0.53, 1.44 | 2.34a | 1.21, 3.46 |
| All-cause mortality | Never smoker | --- | --- | --- | --- | --- | --- |
|  | Long time former smoker | 1.58a | 0.21, 2.95 | 0.20a | 0.0047, 0.41 | 1.78a | 0.41, 3.14 |
|  | Recent former smoker | 3.68a | 0.94, 6.42 | 0.84a | 0.021, 1.67 | 4.52a | 1.86, 7.16 |
|  | Current smoker | 8.52a | 5.76, 11.3 | 1.91a | 0.060, 3.75 | 10.4a | 8.27, 12.6 |

1. 95% CI: 95% confidence interval; 2) a denotes *P* <0.05.

Table S2a. Mediation analysis with AFT model, with pack-years as exposure, data from n=3523 subjects. The effects are presented as fold change of survival time per 1 pack-year.

| Aimed event | Direct effect | | Indirect effect | | Total effect | |
| --- | --- | --- | --- | --- | --- | --- |
|  | estimate | 95% CI | estimate | 95% CI | estimate | 95% CI |
| Acute coronary event | 1.0003 | 0.9957, 1.0050 | 0.9970a | 0.9949, 0.9990 | 0.9973 | 0.9933, 1.0013 |
| Major adverse coronary event | 0.9995 | 0.9954, 1.0035 | 0.9962a | 0.9944, 0.9980 | 0.9957a | 0.9922, 0.9992 |
| Stroke | 1.0016 | 0.9957, 1.0075 | 0.9966a | 0.9941, 0.9990 | 0.9982 | 0.9932, 1.0032 |
| Cardiovascular mortality | 0.9997 | 0.9986, 1.0009 | 0.9990a | 0.9985, 0.9994 | 0.9987a | 0.9977, 0.9997 |
| All-cause mortality | 0.9995 | 0.9988, 1.0002 | 0.9987a | 0.9985, 0.9990 | 0.9982a | 0.9977, 0.9988 |

1. 95% CI: 95% confidence interval; 2) a denotes *P* <0.05.

Table S2b. Mediation analysis with Aalen model, with pack-years as exposure, data from n=3523 subjects. The effects are presented as number of additional cases per 1000 person-years per 1 pack-year.

| Aimed event | Direct effect | | Indirect effect | | Total effect | |
| --- | --- | --- | --- | --- | --- | --- |
|  | estimate | 95% CI | estimate | 95% CI | estimate | 95% CI |
| Acute coronary event | 0.0063 | -0.0329, 0.0455 | 0.0180a | 0.0021, 0.0342 | 0.0243 | -0.0088, 0.0571 |
| Major adverse coronary event | 0.0173 | -0.0275, 0.0621 | 0.0272a | 0.0059, 0.0487 | 0.0446a | 0.0064, 0.0828 |
| Stroke | -0.0051 | -0.0306, 0.0205 | 0.0128a | 0.0008, 0.0248 | 0.0077 | -0.0156, 0.0310 |
| Cardiovascular mortality | 0.0087 | -0.0212, 0.0386 | 0.0183a | 0.0056, 0.0311 | 0.0271a | 0.0000, 0.0540 |
| All-cause mortality | 0.0404 | -0.0195, 0.1003 | 0.0832a | 0.0568, 0.1106 | 0.1235a | 0.0679, 0.1789 |

1. 95% CI: 95% confidence interval; 2) a denotes *P* <0.05.

Table S3a. Mediation analysis with AFT model including 515 participants with inconsistent reported smoking status, data from n=4819 subjects. The effects are presented as fold change of survival time.

| Aimed event | Smoking status | Direct effect | | Indirect effect | | Total effect | |
| --- | --- | --- | --- | --- | --- | --- | --- |
|  |  | estimate | 95% CI | estimate | 95% CI | estimate | 95% CI |
| Acute coronary event | Never smoker | --- | --- | --- | --- | --- | --- |
|  | Long time former smoker | 1.05 | 0.89, 1.23 | 1.00 | 0.99, 1.00 | 1.04 | 0.89, 1.23 |
|  | Recent former smoker | 0.88 | 0.68, 1.13 | 0.99 | 0.96, 1.02 | 0.87 | 0.67, 1.12 |
|  | Current smoker | 0.81a | 0.67, 0.99 | 0.96 | 0.87, 1.07 | 0.78a | 0.67, 0.92 |
| Major adverse coronary event | Never smoker | --- | --- | --- | --- | --- | --- |
|  | Long time former smoker | 0.98 | 0.84, 1.14 | 1.00 | 0.99, 1.00 | 0.97 | 0.84, 1.13 |
|  | Recent former smoker | 0.86 | 0.68, 1.09 | 0.97 | 0.95, 1.00 | 0.84 | 0.66, 1.06 |
|  | Current smoker | 0.84 | 0.70, 1.00 | 0.91 | 0.83, 1.00 | 0.76a | 0.66, 0.89 |
| Stroke | Never smoker | --- | --- | --- | --- | --- | --- |
|  | Long time former smoker | 1.13 | 0.94, 1.37 | 1.00 | 0.99, 1.00 | 1.13 | 0.93, 1.37 |
|  | Recent former smoker | 0.87 | 0.65, 1.15 | 0.98 | 0.94, 1.01 | 0.85 | 0.64, 1.12 |
|  | Current smoker | 0.87 | 0.70, 1.09 | 0.91 | 0.81, 1.03 | 0.80a | 0.67, 0.95 |
| Cardiovascular mortality | Never smoker | --- | --- | --- | --- | --- | --- |
|  | Long time former smoker | 0.96 | 0.82, 1.11 | 1.00 | 0.99, 1.00 | 0.96 | 0.82, 1.11 |
|  | Recent former smoker | 0.88 | 0.69, 1.11 | 0.99 | 0.96, 1.01 | 0.87 | 0.69, 1.09 |
|  | Current smoker | 0.80a | 0.67, 0.96 | 0.96 | 0.87, 1.06 | 0.77a | 0.66, 0.89 |
| All-cause mortality | Never smoker | --- | --- | --- | --- | --- | --- |
|  | Long time former smoker | 0.93 | 0.84, 1.02 | 1.00 | 0.99, 1.00 | 0.92 | 0.84, 1.02 |
|  | Recent former smoker | 0.82a | 0.71, 0.95 | 0.97a | 0.96, 0.99 | 0.80a | 0.69, 0.92 |
|  | Current smoker | 0.78a | 0.70, 0.87 | 0.90a | 0.85, 0.95 | 0.70a | 0.64, 0.77 |

1. 95% CI: 95% confidence interval; 2) a denotes *P* <0.05.

Table S3b. Mediation analysis with Aalen model including 515 participants with inconsistent reported smoking status, data from n=4819 subjects. The effects are presented as number of additional cases per 1000 person-years.

| Aimed event | Smoking status | Direct effect | | Indirect effect | | Total effect | |
| --- | --- | --- | --- | --- | --- | --- | --- |
|  |  | estimate | 95% CI | estimate | 95% CI | estimate | 95% CI |
| Acute coronary event | Never smoker | --- | --- | --- | --- | --- | --- |
|  | Long time former smoker | 0.030 | -0.85, 0.91 | 0.0076 | -0.023, 0.044 | 0.037 | -0.85, 0.92 |
|  | Recent former smoker | 1.37 | -0.40, 3.14 | 0.054 | -0.15, 0.26 | 1.43 | -0.35, 3.19 |
|  | Current smoker | 1.31a | 0.12, 2.50 | 0.19 | -0.53, 0.93 | 1.50a | 0.53, 2.47 |
| Major adverse coronary event | Never smoker | --- | --- | --- | --- | --- | --- |
|  | Long time former smoker | 0.47 | -0.56, 1.50 | 0.030 | -0.0088, 0.091 | 0.50 | -0.52, 1.53 |
|  | Recent former smoker | 1.85 | -0.20, 3.90 | 0.22 | -0.056, 0.50 | 2.07a | 0.011, 4.11 |
|  | Current smoker | 1.28 | -0.14, 2.70 | 0.79 | -0.20, 1.79 | 2.07a | 0.96, 3.17 |
| Stroke | Never smoker | --- | --- | --- | --- | --- | --- |
|  | Long time former smoker | -0.40 | -1.24, 0.44 | 0.022 | -0.0068, 0.067 | -0.38 | -1.22, 0.46 |
|  | Recent former smoker | 0.95 | -0.73, 2.62 | 0.15 | -0.045, 0.35 | 1.10 | -0.58, 2.78 |
|  | Current smoker | 0.54 | -0.58, 1.66 | 0.54 | -0.16, 1.23 | 1.08a | 0.15, 2.01 |
| Cardiovascular mortality | Never smoker | --- | --- | --- | --- | --- | --- |
|  | Long time former smoker | 0.37 | -0.33, 1.06 | 0.0073 | -0.018, 0.038 | 0.37 | -0.32, 1.07 |
|  | Recent former smoker | 0.90 | -0.47, 2.27 | 0.048 | -0.11, 0.21 | 0.95 | -0.41, 2.30 |
|  | Current smoker | 1.07a | 0.13, 2.01 | 0.18 | -0.40, 0.76 | 1.25a | 0.46, 2.03 |
| All-cause mortality | Never smoker | --- | --- | --- | --- | --- | --- |
|  | Long time former smoker | 1.32a | 0.010, 2.63 | 0.10a | 0.011, 0.22 | 1.42a | 0.10, 2.73 |
|  | Recent former smoker | 3.49a | 0.88, 6.1 | 0.67a | 0.29, 1.09 | 4.15a | 1.57, 6.74 |
|  | Current smoker | 3.19a | 1.41, 4.97 | 2.46a | 1.12, 3.79 | 5.64a | 4.13, 7.15 |

1. 95% CI: 95% confidence interval; 2) a denotes *P* <0.05.

Table S4. Sensitivity analysis for unmeasured mediator-outcome confounder. The results of direct effects and indirect effects for major adverse cardiac event in current smoker group were taken as an example here. The analysis is made for the situation where the unmeasured confounder increases cadmium (α3>0) and shortens survival time (exp(β5)<1, AFT) or increases the hazard (β5>0, Aalen). Corrected estimates of direct and indirect effects are listed below, given certain values of the effect of the unmeasured confounder on cadmium (α3), the effect of the confounder on survival (exp(β5) for AFT model and β5 for Aalen model).

| Survival Model: AFT model | | | |
| --- | --- | --- | --- |
| α3 | exp(β5) | Direct effect | Indirect effect |
| No confounding | | | |
| 0 | 1 | 0.86 | 0.85 |
| The unmeasured confounder increases cadmium and shortens survival time | | | |
| 0.2 | 0.95 | 0.796 | 0.919 |
| 0.2 | 0.92 | 0.758 | 0.965 |
| 0.2 | 0.88 | 0.708 | 1.032 |
| 0.3 | 0.95 | 0.77 | 0.96 |
| 0.3 | 0.92 | 0.71 | 1.03 |
| 0.3 | 0.88 | 0.64 | 1.14 |
| 0.4 | 0.95 | 0.74 | 0.99 |
| 0.4 | 0.92 | 0.67 | 1.09 |
| 0.4 | 0.88 | 0.58 | 1.25 |
| Survival Model: Aalen model | | | |
| α3 | β5 | Direct effect | Indirect effect |
| No confounding | | | |
| 0 | 0 | 1.10 | 1.50 |
| The unmeasured confounder increases cadmium and increases hazard | | | |
| 0.2 | 0.3 | 1.56 | 1.04 |
| 0.2 | 0.5 | 1.86 | 0.74 |
| 0.2 | 0.7 | 2.16 | 0.44 |
| 0.3 | 0.3 | 1.78 | 0.82 |
| 0.3 | 0.5 | 2.24 | 0.36 |
| 0.3 | 0.7 | 2.69 | -0.09 |
| 0.4 | 0.3 | 2.01 | 0.59 |
| 0.4 | 0.5 | 2.62 | -0.02 |
| 0.4 | 0.7 | 3.22 | -0.62 |

1) Notations used are: (β_0_ + β_1_∙Exp + β_2_∙Med + β_3_∙Exp∙Med + β_4_∙C + β_5_∙U) for the outcome regression model, and (α_0_+ α_1_∙Exp + α_2_∙C + α_3_∙U) with residual variance σ^2^_M_ for the linear mediator regression model. The parameters used in this analysis (values for α_1_ and σ^2^_M_) are derived from the models. In this example, α_1_=0.88 and σ^2^_M_=0.116.
